# Abiotic stresses influence the transcript abundance of PIP and TIP aquaporins in *Festuca* species

**DOI:** 10.1007/s13353-017-0403-8

**Published:** 2017-08-04

**Authors:** Izabela Pawłowicz, Marcin Rapacz, Dawid Perlikowski, Krzysztof Gondek, Arkadiusz Kosmala

**Affiliations:** 1 Institute of Plant Genetics, Polish Academy of Sciences, Strzeszynska 34, 60-479 Poznan, Poland; 2 Department of Plant Physiology, Faculty of Agriculture and Economics, University of Agriculture in Krakow, Podluzna 3, 30-239 Krakow, Poland; 3 Department of Agricultural and Environmental Chemistry, University of Agriculture in Krakow, Aleja Mickiewicza 21, 31-120 Krakow, Poland

**Keywords:** Aquaporin, *Festuca arundinacea*, *Festuca pratensis*, Real time qRT-PCR, Transcript, Stress tolerance

## Abstract

*Festuca arundinacea* and *F. pratensis* are the models in forage grasses to recognize the molecular basis of drought, salt and frost tolerance, respectively. Transcription profiles of plasma membrane intrinsic proteins (PIPs) and tonoplast intrinsic proteins (TIPs) aquaporin genes were obtained for leaves of *Festuca* species treated with different abiotic stimuli. *F. arundinacea* plants were exposed to drought and salt stress, whereas *F. pratensis* plants were cold-hardened. Changes in genes expression measured with use of real time qRT-PCR method were compared between two genotypes characterized with a significantly different level of each stress tolerance. Under drought the transcript level of PIP1;2 and TIP1;1 aquaporin decreased in both analyzed *F. arundinacea* genotypes, whereas for PIP2;1 only in a high drought tolerant plant. A salt treatment caused a reduction of PIP1;2 transcript level in a high salt tolerant genotype and an increase of TIP1;1 transcript abundance in both *F. arundinacea* genotypes, but it did not influence the expression of PIP2;1 aquaporin. During cold-hardening a decrease of PIP1;2, PIP2;1, and TIP1;1 aquaporin transcripts was observed, both in high and low frost tolerant genotypes. The obtained results revealed that the selected genotypes responded in a different way to abiotic stresses application. A reduced level of PIP1;2 transcript in *F. arundinacea* low drought tolerant genotype corresponded with a faster water loss and a lowering of photosynthesis efficiency and gas exchange during drought conditions. In *F. pratensis*, cold acclimation was associated with a lower level of aquaporin transcripts in both high and low frost tolerant genotypes. This is the first report on aquaporin transcriptional profiling under abiotic stress condition in forage grasses.

**Electronic supplementary material:**

The online version of this article (doi:10.1007/s13353-017-0403-8) contains supplementary material, which is available to authorized users.

## Introduction

The most severe factors that affect the growth and reduce the yield of crops worldwide are drought, salinity, and low temperature. Plants have evolved different kinds of strategies leading to adaptation to these abiotic stresses (Fujita et al. 2006), and maintaining water homeostasis is one of the most important. Water uptake from soil to roots, and its subsequent relocation to the aerial parts of the plant, are the critical steps limiting the efficiency of its usage (WUE – water use efficiency) in physiological processes (Chaumont and Tyerman 2014); and the main role in this water movement is played by aquaporins. These are proteins that constitute water channels in biological membranes, and take part in symplastic and transcellular pathways of the flow of water molecules within the plant body.

Aquaporins belong to a family of highly conserved major intrinsic proteins (MIP), that have representatives in all living organisms (archaea, eubacteria, fungi, plants, and animals) (Gomes et al. 2009). They are involved in bidirectional flux of water and small neutral solutes through biological membranes. In the plant kingdom a surprisingly large number of them have been found. They range from 33 in *Zea mays* L. (Chaumont et al. 2001), 35 in *Arabidopsis thaliana* L. (referred here as *Arabidopsis*) (Johanson et al. 2001) and to more than 70 in *Gossypium hirsutum* L. (Park et al. 2010). The multiplicity of plant aquaporin isoforms suggests they have a highly important and complicated role in water relations, and an involvement in several different cellular processes.

Based on sequence similarity and subcellular localization plant aquaporins are classified into five sub-families: plasma membrane intrinsic proteins (PIPs), tonoplast intrinsic proteins (TIPs), nodulin26 (Nod26)-like intrinsic proteins (NIPs), small basic intrinsic proteins (SIPs) (Chaumont et al. 2001; Ishibashi et al. 2011; Maurel et al. 2015), and X intrinsic proteins (XIPs) (Bienert et al. 2011; Kammerloher et al. 1994). PIPs and TIPs are the most abundant in plasma membrane and in the tonoplast, respectively. NIPs aquaporins were initially identified in the symbiosomes of legumes, but are also localized in the plasma membrane and endoplasmic reticulum. The XIPs subfamily has been found in nonvascular and vascular plants, but it lacks representatives in the *Brassicaceae* and in monocots (Borstlap 2002; Danielson and Johanson 2008). Among the PIPs sub-family two groups of isoforms can be distinguished, PIP1 and PIP2 (Chaumont et al. 2000), with PIP2 having a higher water channel activity than PIP1.

It is strongly suggested that PIPs take part in a trans-cellular water transport, whereas TIPs are involved in an osmotic adjustment and take part in the regulation of water exchange between the cytosolic and vacuolar compartments. Except for their participation in water movement across biological membranes, aquaporins are also involved in the transportation of different molecules such as small uncharged solutes (glycerol, urea, ammonia, hydrogen peroxide, boric acid) (Dordas et al. 2000; Gerbeau et al. 1999; Henzler and Steudle 2000; Liu et al. 2003) and gases (carbon dioxide) (Katsuhara and Hanba 2008). A different kinds of transporting molecules and the tissue localization of particular aquaporins are tightly associated with their physiological role (Alexandersson et al. 2005; Besse et al. 2011). They are involved in such processes as cell division and expansion, carbon fixation, acquisition of nutrients, and cell signaling (Maurel et al. 2015).

It has been revealed that PIPs isoforms can facilitate not only water, but also carbon dioxide transport through membranes (Heckwolf et al. 2011; Uehlein et al. 2003), which strongly indicates that the involvement of aquaporins in water fluxes in plants determines their regulative role in CO_2_ assimilation through stomata. The contribution of PIPs to mesophyll conductance (*g*_m_) consequently has an impact on photosynthesis rates (Sade et al. 2014). Analysis of tobacco, rice, and *Arabidopsis* transgenic plants, in well-watered conditions, shows that decline of aquaporin accumulation level caused net CO_2_ (*A*_net_) assimilation suppression, whereas in overexpressing lines *A*_net_ was elevated (Flexas et al. 2006; Heckwolf et al. 2011; Kawase et al. 2013). This suggests that in photosynthesis limitation *g*_m_ has a bigger contribution, compared to stomatal conductance (*g*_s_).

There have also been reports that aquaporin genes respond to abiotic stimuli like drought, high salinity or low temperature. Changes in the gene expression at the transcriptional level during abiotic stresses were observed in such species as *Arabidopsis* (Jang et al. 2004; Wang et al. 2015a, b), *Triticum aestivum* L. (Huang et al. 2014), *Vitis vinifera* L. (Pou et al. 2013) and *Hordeum vulgare* L. (Hove et al. 2015). Particular isoforms of aquaporin showed up- and down-regulation, depending on the type of stress and its duration. It is also suggested that expression patterns of particular aquaporins can fluctuate during stress treatment, and differ in response to short and long stress, which is associated with releasing various defense responses. Many studies indicate that at the beginning of stress response, plants reduce the accumulation/activity of aquaporins to save the water content. During long-term stress a higher accumulation, or activity, of aquaporins was observed, which allows compensation for water deficiencies (Chaumont and Tyerman 2014) taking place in the plant after a longer stress treatment. Variant roles of particular aquaporins in abiotic stress tolerance strategies can be observed in transgenic plants. Elevated or decreased level of particular genes expression can have both a beneficial or negative impact on plant stress tolerance, resulting in changes in a root hydraulic conductivity, transpiration rates, cellular osmotic potential, and plant ability to recover from water deficit stress (Ahamed et al. 2012; Cui et al. 2008; Khan et al. 2015; Laur and Hacke 2014; Peng et al. 2007; Wang et al. 2015a, b).

Tall fescue (*Festuca arundinacea* Schreb.; Fa) and meadow fescue (*Festuca pratensis* Huds.; Fp) are important forage grass species in temperate regions. They are commonly utilized as model plants in studies relating to molecular mechanisms underlying abiotic stress tolerance (Alm et al. 2011; Kim et al. 2010; Kosmala et al. 2009, 2012; Ma et al. 2014). *F. arundinacea* is characterized by superior drought, heat, and salt tolerance (Mian et al. 2008; Wang and Ge 2006), whereas *F. pratensis* exhibits a high ability to cold acclimation, and consequently to high frost tolerance (Kosmala et al. 2009). The role of aquaporins in the abiotic stress response in *planta* still remains a matter of debate. In this paper, we present the expression profiles of PIPs and TIPs aquaporins in model grass species under drought and high salinity conditions in *F. arundinacea* and under cold acclimation conditions in *F. pratensis*. In both species two genotypes differing in the levels of stress tolerance were applied into the research, high and low drought as well as high and low salt-tolerant genotypes of *F. arundinacea* and high and low frost tolerant genotypes of *F. pratensis*. The studies were performed to decipher the impact of the different abiotic stress factors on the expression level of individual aquaporins from PIPs and TIPs sub-families. Additionally we tried to find out if the transcript abundance differed between stress susceptible and stress tolerant genotypes of *F. arundinacea* and *F. pratensis* and to formulate some conclusions regarding aquaporins involvement in stress tolerance in forage grasses.

## Material and methods

### Plant material

The expression of *Pip1;2*, *Pip2;1*, *Tip1;1*, and *Tip2;1* aquaporin genes was profiled in the plants subjected to drought, salinity and cold conditions. A separate analysis was performed on two genotypes differentiated with respect to their tolerance level to each analyzed stress: (*i*) high and low drought tolerant *F. arundinaca* (Fa45 - HDT and Fa60 - LDT, respectively), selected by Pawłowicz and Rapacz (2010), and analyzed with respect to the physiological parameters by Kosmala et al. (2012); (*ii*) high and low salt tolerant *F. arundinacea* (Fa18 - HST and Fa14 - LST, respectively), selected and physiologically analyzed in this paper; and (*iii*) high and low frost tolerant (Fp37 - HFT and Fp13 - LFT, respectively), selected by Kosmala et al. (2009) (Table 1).

**Table 1:** Abbreviations and numbers of genotypes selected from *F. arundinacea* population treated with drought and salinity and *F. pratensis* population treated with low temperature

*Stress*	*Low stress tolerant genotype*	*High stress tolerant genotype*	*Species*
Drought	LDT (Fa60)	HDT (Fa45)	*Festuca arundinacea*
Salinity	LST (Fa14)	HST (Fa18)	*Festuca arundinacea*
Low temperature	LFT (Fp13)	HFT (Fp37)	*Festuca pratensis*

*LDT* low drought tolerant, *HDT* high drought tolerant, *LST* low salt tolerant, *HST* high salt tolerant, *LFT* low frost tolerant, *HFT* high frost tolerant

### Stress experiment conditions

#### Drought

Conditions of the drought experiment were described in detail by Kosmala et al. (2012). Equal-sized clones of each genotype were planted into pots and placed into a growth chamber with the following regime: temperature – 22/17 °C, photoperiod – 16 h day/8 h night, light – 400 μmol m^−2^ s^−1^, HPS ‘Agro’ LAMPS, Philips, Brussels, relative air humidity – 30%. Watering of LDT and HDT plants was then withheld. Material was collected at six time points: before drought (0D), 3, 6, 9, and 11 days of water deficit (3-11D) and irrigation following stress treatment (RE). At each time point measurements of physiological parameters (RWC, EL, chlorophyll fluorescence, soil water content) were performed as described by Kosmala et al. (2012).

#### Salinity

**Fig. 1:**
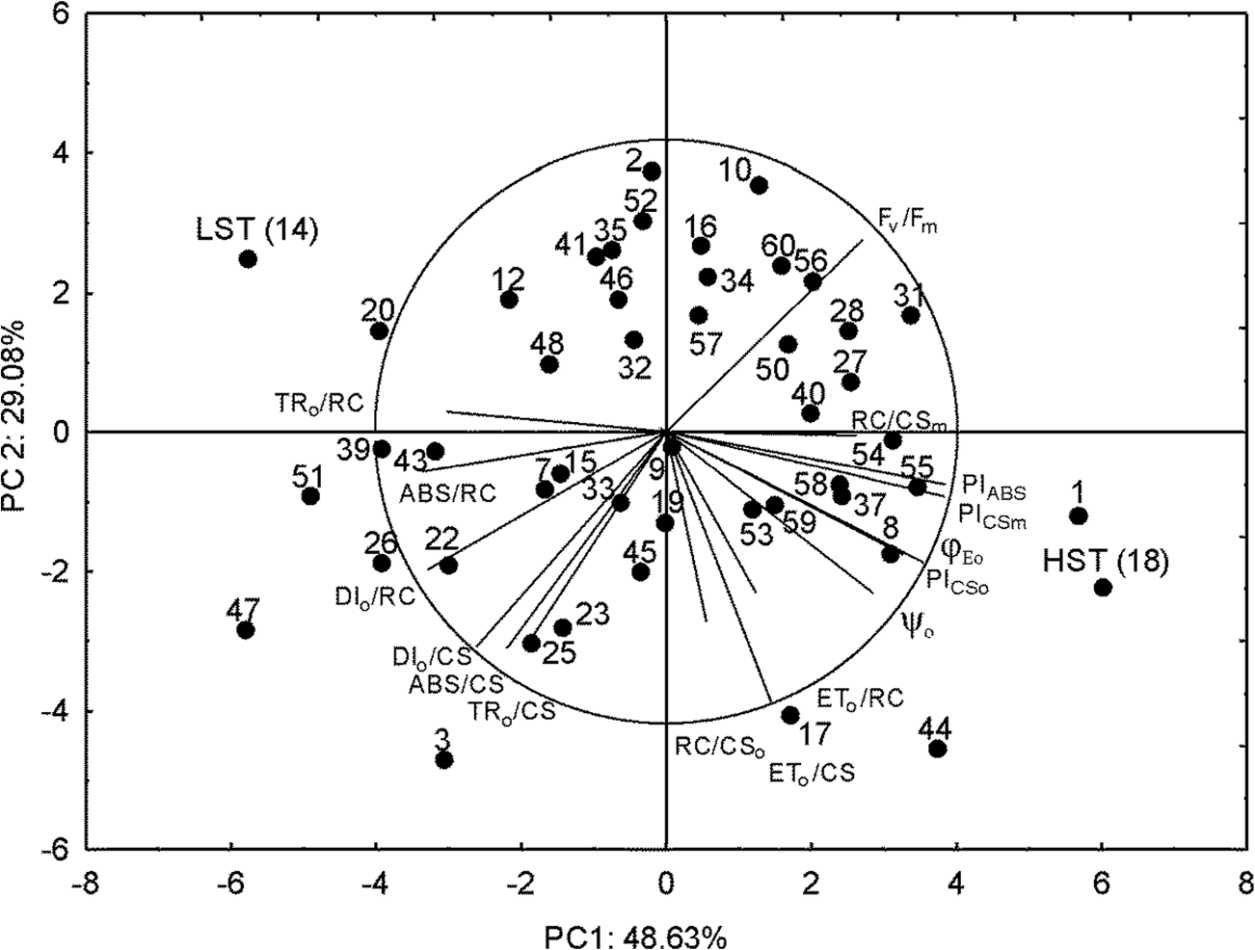
Biplot PCA discrimination of different response of PSII performance in the studied genotypes of *F. arundinacea* to salinity. Variables represented the changes of OJIP test parameters under conditions of salinity related to the control conditions. Unit *circle* is presented in 4 × magnified scale. High salt tolerant (HST) and low salt tolerant (LST) genotypes were indicated. The original numbers of genotypes are also indicated

*F. arundinacea* population cv. Kord consisting of 47 genotypes was evaluated with respect to salt tolerance by chlorophyll fluorescence measurements. Genotypes (obtained from single seeds) at approximately 4 months old were cloned into six plants (three for the control and three for NaCl treatment) with 3–5 tillers each, and planted into pots (20 cm in diameter, 3.5 dm^3^ in volume). The plants were then placed into the growth chamber with the following regime: temperature – 22/17 °C, photoperiod – 16 h day/8 h night, light – 400 μmol m^−2^ s^−1^, HPS ‘Agro’ LAMPS, Philips, Brussels, relative air humidity – 60%. Salt stress was performed after three weeks by irrigating plants with 250 mM NaCl solution instead of water. Before NaCl application the water status in each pot was aligned to the value of 100% field water capacity. Plants were then irrigated with 200 cm^3^ of NaCl every 2 days through to 21 days. The experiment was performed on 47 genotypes where no visual symptoms of depleted growth after cloning was observed in all six clones. Measurements of chlorophyll fluorescence in the control conditions and after salt treatment were then taken. On the basis of change in the contrasting OJIP parameters plants were differentiated with respect to salt tolerance within the analyzed population. Two genotypes: high salt tolerant (HST) and low salt tolerant (LST) were selected (Fig. 1).

Two selected genotypes were stress-treated again. Plant material was collected at the following time points: before salt treatment (0 NaCl), 3, 6, 9, and 11 days of salt treatment (3–11 NaCl). At each time-point measurements of relative water content (RWC) in leaves, sodium, and potassium content and chlorophyll fluorescence were performed.

#### Low temperature

The freezing tolerance of LFT and HFT genotypes was determined by performing the *T*_EL50_ test (temperature causing a 50% electrolyte leakage) after 21 days of cold acclimation (Kosmala et al. 2009), and this revealed that the Fp13 (LFT) genotype reached *T*_EL50_ value at −15.9 °C temperature, whereas Fp37 (HFT) reached it at −21.4 °C (Kosmala et al. 2009).

A procedure of low temperature pre-hardening and hardening was published by Kosmala et al. (2009). The clones of HFT and LFT *F. pratensis* genotypes were put into a growth chamber for pre-hardening for 7 days (temperature – 12 °C photoperiod – 8 h day/16 h night, light 200 μmol m^−2^ s^−1^ PPFD). Next, the plants were cold-acclimated for 21 days under the following conditions: temperature – 4/2 °C, photoperiod – 10 h day/14 h night, light 200 μmol m^−2^ s^−1^ PPFD). The plant material was collected at the following time points: before hardening (0CA), 3, 5, 7, 14, and 21 days of cold (3-21CA).

### Sample collection

Leaves at the individual time points of each stress treatment were collected in three biological replicates, immediately frozen in liquid nitrogen, and stored at −80 °C.

### Chlorophyll fluorescence measurements and OJIP analysis

Chlorophyll fluorescence measurements were performed using the Handy PEA fluorimeter (Hansatech). Measurements were taken with a 3000 μmol(quanta) m^−2^ s^−1^ saturated excitation light after 30 min of dark adaptation in leaf clips. Changes in the chlorophyll fluorescence signal were registered between 10 μs and 1 s of saturated light pulse.

OJIP test parameters were calculated using the generated chlorophyll fluorescence induction curve (Srivastava et al. 1995; Strasser et al. 2000). The calculation includes: specific energy fluxes for single PSII reaction centers (RCs): ABS/RC (for absorbed energy), TRo/RC (for trapped energy), ETo/RC (for electron transport), and DIo/RC (for dissipated energy). Phenomenological energy fluxes were calculated for the area of the photosynthetic sample (CS) at *t* = 0 as follows: ABS/CS (for absorbed energy), TRo/CS (for trapped energy), ETo/CS (for electron transport), and DIo/CS (for dissipated energy). Calculations also included: performance indexes on a CS basis (PICS) in relaxed and excited states of PSII (PICSo and PICSm, respectively), performance index based on absorbed energy amount (PIABS) and densities of active PSII reaction centers at *t* = 0 and tmax (time to reach maximum fluorescence): RC/CSo and RC/CSm, respectively. Yield ratios were calculated for *t* = 0: maximum quantum yield of primary photochemistry (φPo or Fv/Fm), quantum yield of electron transport (*φ*Eo), and the overall quantum yield between light absorption and the electron transport beyond quinone A (*ψ*o). Detailed calculations for OJIP parameters are listed in Rapacz et al. (2015).

The measurements were performed on young, fully expanded leaves at midday in nine replicates.

### RWC measurement

The RWC in leaves was calculated using the following formula: RWC(%) = (FW-DW)/(SW-DW) × 100, where FW was leaf fresh weight, DW was leaf dry weight and SW was leaf turgid weight. Leaves were first cut into segments, and then immediately weighed (FW). After overnight submergence in tubes with sterile water leaves were weighed again (SW). Leaves were then dried (70 °C, 2 days) and the DW value was obtained.

### Sodium and potassium content measurement

The contents of potassium and sodium in *F. arundinacea* leaves during control conditions, salt treatment, and recovery were determined after incinerating the sample in a chamber furnace at 450 °C for 12 h. The remains were dissolved in diluted nitric acid 1:2 (*v*/v) (Oleszczuk et al. 2007). The concentration of the elements was determined by an inductively coupled plasma optical emission spectrometry (ICP-OES, Perkin Elmer Optima 7300 DV).

### The cloning of aquaporin cDNA sequences from *F. arundinacea*

Full length cDNA sequences encoding the aquaporins—PIP1;2, PIP2;1, TIP1;1, and TIP2;1—were obtained from the *Festuca arundinacea* genome using a PCR. Primers were designed based on *Lolium perenne* sequences obtained by sequencing its genome (Byrne et al. 2015). The open reading frames (ORF) for individual genes were as follows: 759 bp for FaTIP1;1, 750 bp for FaTIP2;1, 867 bp for FaPIP1;2 and 864 bp for FaPIP2;1 (Online Resource 1).

The phylogenetic relationship between *F. arundinacea* aquaporin genes and the genes from different plant species was shown by creating phylogenetic trees for each gene (Online Resource 2). The *F. arundinacea* genome consists of three sub-genomes: one from *F. pratensis* and two from *F. glaucescens* (Humphreys et al. 1995). For this reason, aquaporin gene cloning was performed on *F. arundinacea*. For expression profiling with use of real time PCR, one pair of primers for each gene was designed and they generated single PCR products both in *F. arundinacea* and *F. pratensis* species. Subsequent cloning of these PCR products confirmed sequenced similarity with desired genes.

The resulting PCR products were purified using QIAEXII Gel Extraction Kit (Qiagen) and ligated into the pGEM-T Easy vector (Promega). Next, *Escherichia coli* strain XL1 Blue was transformed with the ligation mixture. The selected X-Gal and IPTG clones carrying an appropriate PCR product were sequenced (Molecular Biology Techniques Laboratory, Faculty of Biology, Adam Mickiewicz University, Poznań). The obtained sequences were processed with BioEdit software.

### RNA extraction and qRT-PCR performance

Total RNA was extracted from *F. arundinacea* and *F. pratensis* leaves using Total RNA Purification Kit (Novazym, Poland), and then treated with DNAse I (Roche). Next, cDNA was synthesized from 1 μg of RNA (Trasnscriptor First Strand cDNA Synthesis Kit (Roche), according to the enclosed protocol.

Quantitative real-time PCR assays were performed in the Bio-Rad CFX 96 thermal system. Reaction mixture of final 10 μl volume contained 100–300 nM of each primer, 1 μl of cDNA, and 5 μl of AmpliQ Real Time Opti Probe Kit (Novazym, Poland). All the PCR reactions were performed using the following parameters: 95 °C for 10 min, 44 cycles of 95 °C for 15 s, and 60 °C for 30 s. For real-time reactions a normalization with two reference genes were used: actin and ubiquitin. Primers and TaqMan probe sequences for real time qRT-PCR designed using Beacon Designer software, amplicon length, and reaction efficiency of particular gene analyzed are given in Table 2. An expression stability of reference genes under the stress conditions was calculated by BestKeeper (Online Resource 3). The data was analyzed using the relative quantification method. All the assays were performed with three biological and two technical replicates.

**Table 2:** Primer and TaqMan probe sequences, amplicon length, and PCR efficiency of aquaporin (PIP1;2, PIP2;1, TIP1;1, and TIP2;1) and reference genes (actin – AKT and ubiquitin – UBQ) in *F. arundinacea* and *F. pratensis* samples

	Primer sequence	TaqMan sequence	Amplicon length (bp)	PCR efficiency (*F. arundinacea*) (%)	PCR efficiency (*F. pratensis*) (%)
*PIP1;2*	GGTGTAGACGAGGACGAAGG GGCGGTGTTCTACATCGTG	CTTGGTGTAGCCGGCGTTCA	180	101.0	96.1
*PIP2;1*	CGTCCTCGTCTACACCGTCT CACCCAGAAGATCCATTGGT	CGAGAAGCCTGGGAGCTGCG	203	100.7	100.0
*TIP1;1*	GAACCACTGGGTGTACTG GCCGATGAAGATGATGTC	CCGCCATCGCCGCGCTCAT	79	102.2	100.0
*TIP2;1*	CCAGATCACCATCCTCAC CGTACACGGTGTAAACGA	ATGACCTCCATCACGACGCC	191	–	–
*AKT*	GTCGAGGGCAACATATGCAA CCAGTGCTGAGCGGGAAT	TTCTCCTTGATGTCACGGAC	65	100.1	100.9
*UBQ*	GCAAGAAGAAGACGTACA GACCTTGTAGAACTGGAG	CTTCACCTTCTTGTCCTTGTGCTT	86	98.8	98.3

### Statistical analysis

All the statistical analysis was performed with Statistica 12 software (StatSoft, Tulsa, OK). The results concerning chlorophyll fluorescence measurements under salt stress were subjected to a GLM analysis, with genotype and treatment as factors. The significance of differences between means was estimated using Tukey’s HSD test. Principal component analysis (PCA) of OJIP-test data was used to discriminate genotypes with contrasting salt tolerance. The PCA was based on eigenvalue decomposition of a data correlation matrix.

The normalized relative transcript expression, RWC, and ion content data was also submitted to statistical analysis. Using two-way ANOVA analysis with genotype and treatment as classification factors, Tukey’s honest significant difference (HSD) was made. The significant effects of genotype, time, and genotype × time interaction were selected using the family-wise error rate less than 5%. Tukey’s HSD of samples at 5% was used (Online Resource 4)*.*

## Results

In the present studies the impact of abiotic stresses on transcript accumulation of four aquaporin genes was analyzed in two fescue species: *F. arundinacea* and *F. pratensis*. The *F. arundinacea* population was treated with drought and salt, whereas the *F. pratensis* population was cold-hardened.

### Plant response to stress at the physiological level

Freezing tolerance of *F. pratensis* plants was estimated based on a regrowth ability after freezing using Larsen’s visual score by Kosmala et al. (2009). The additional criterion to select the most and the least tolerant genotype was also an electrolyte leakage measurement (*T*_EL50_ test, performed before and during hardening to cold). The estimation of *F. arundinacea* genotypes tolerance to drought and salinity, and the selection of the two most contrasting genotypes, were performed based on chlorophyll fluorescence measurements. Details concerning selection (HDT and LDT genotypes) with respect to drought tolerance were described earlier by Kosmala et al. (2012) and by Pawłowicz and Rapacz (2010). The PCA analysis of change in OJIP-test parameters in response to salinity conditions were performed herein and are shown in Fig. 1.

Genotypes Fa18 and Fa14 were selected as high-salinity tolerant and low-salinity tolerant, respectively. The most contrasting parameters for these two genotypes were PSII performance indexes (PI) and the yield of absorbed energy used for electron transport (*φ*Eo). These parameters were additionally most affected by salinity, and showed the highest variation between genotypes (data not shown).

**Fig. 2:**
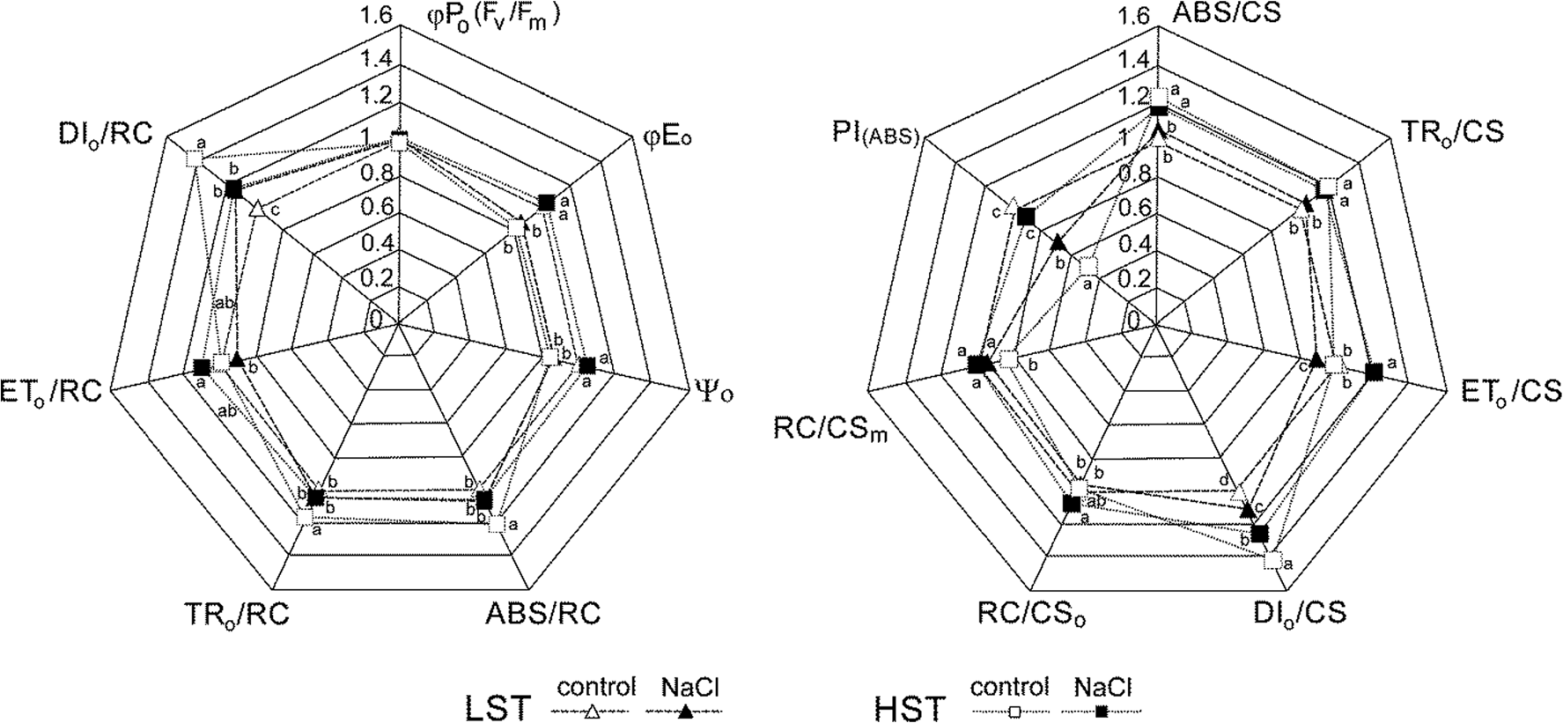
Changes of OJIP-test parameters triggered by salinity treatment in high salt tolerant (HST) and low salt tolerant (LST) genotypes of *Festuca arundinacea*. Values normalized for control values for LST genotype. The *letters* indicated homogeneity groups and *asterisks* indicated the statistical significance of parameter value change under salinity stress (Tukey’s HSD test, *P* = 0.05)

The detailed comparison of PSII performance between the salt-contrasting genotypes are shown in Fig. 2.

Under control conditions the PSII photochemical performance, measured by means of general PSII performance index (PIABS), the yields of photochemical energy conversions (*φ*Eo; *ψ*o), and phenomenological energy flux for electron transfer (ETo/CS) of the LST genotype was higher when compared with the HST. The HST genotype under salinity increased its PSII photochemical performance. The opposite reaction was observed in the LST genotype, and as a result PIABS, *φ*Eo, *ψ*o, and ETo/CS values under salinity were considerable higher in the HST genotype. It is worth mentioning that no differences in maximum quantum yield of PSII (Fv/Fm) were observed between both genotypes and treatments. A low photochemical performance of the control HST plants was connected with high energy dissipation rate in PSII, which was visible for both single, active reaction center (DIo/RC) and unit leaf area (DIo/CS). Also, the number of reactive PSII reaction centers under excited state RC/CSm was lower in the HST than in the LST which, together with no difference in their number under relaxed state (RC/CSo) and differences in energy dissipation observed above, may suggest a higher supply of photoassimilates in the HST control genotype. Under salinity the HST genotype decreased energy dissipation and increased RC/CSm without a significant change in RC/CSo, whereas in the LST genotype energy dissipation increased without any change in the number of active reaction centers.

**Fig. 3:**
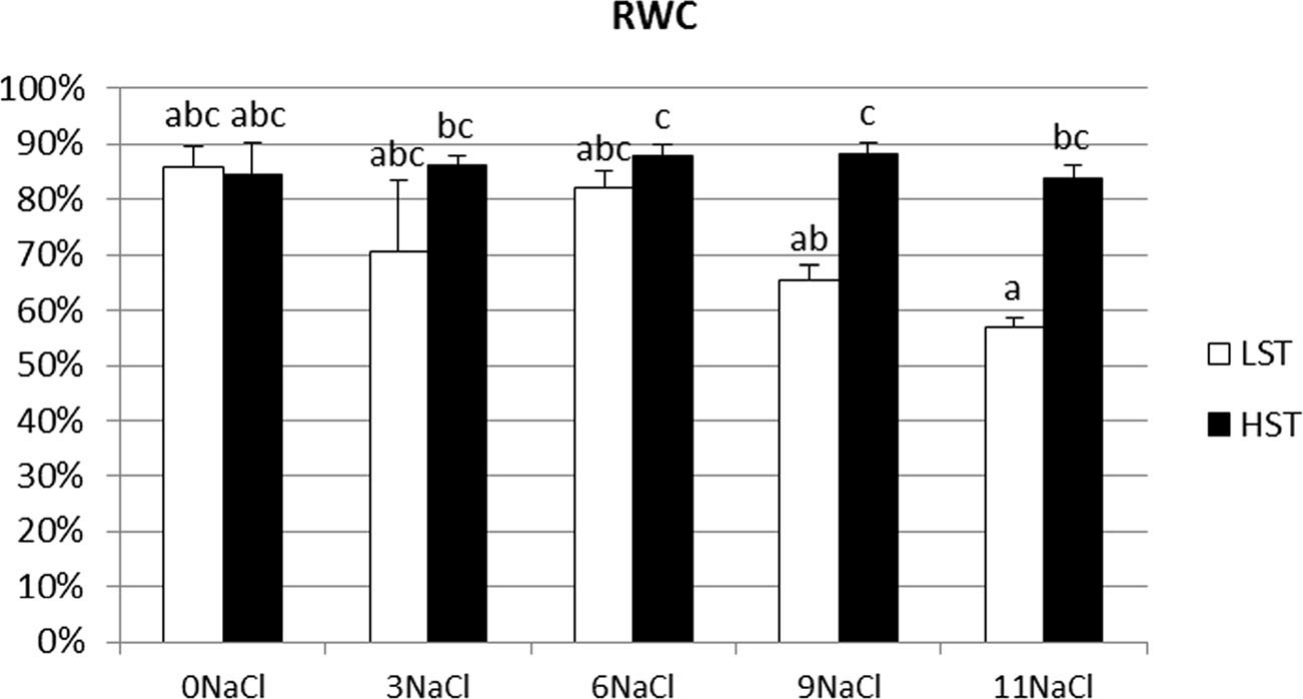
Relative water content (RWC) in *F. arundinacea* LST and HST genotypes under salt stress. Data is mean ± SE calculated from measurements performed for nine individual leaves. Homogeneity groups according to Tukey’s HSD test (*P* < 0.05) are denoted by the same letters

During salt stress RWC values were unchanged in both analyzed genotypes (Fig. 3). Significant differences between the HST and LST plants were observed on the 9th and 11th day of stress treatment.

**Fig. 4:**
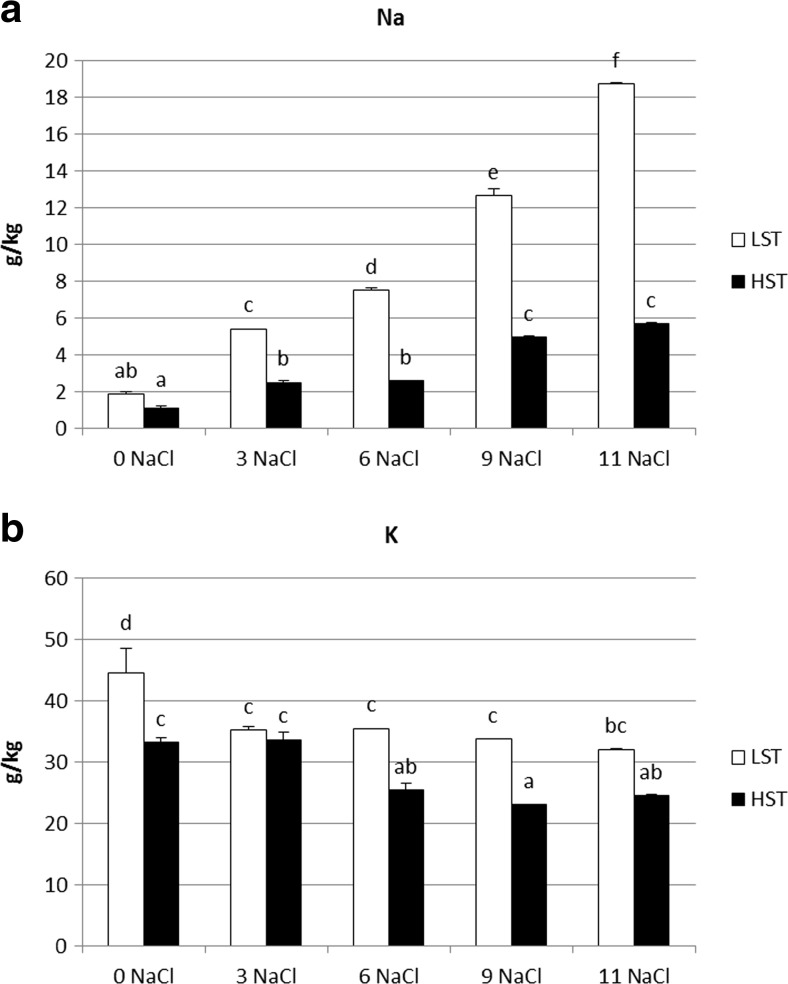
Changes in sodium (**a**) and potassium (**b**) ion concentration in *F. arundinacea* LST and HST genotype under salt stress. The data represent means for nine individual measurements ± standard error (SE). Homogeneity groups according to Tukey’s HSD test (*P* < 0.05) are denoted by the same letters

Sodium ion concentration increased under stress treatment in both genotypes (Fig. 4a). The initial level of sodium was similar in both analyzed genotypes, whereas stress conditions caused it to rise more significantly in the LST plant. In the LST genotype a gradual increase of Na^+^ was observed, giving the highest value on the 11th day of the stress application. In the HST genotype a significant growth of sodium ion concentration was noticed, the first one between the control and the 3rd day, and the second one between the 6th and the 9th day of the stress treatment. Potassium ion concentration decreased slightly in both genotypes during stress (Fig. 4b). In the LST genotype it dropped on day 3 of salt treatment, and stayed at the reduced level during the whole stress period. In the HST genotype reduction of the potassium level was observed between day 6 and 11 of the stress duration with respect to the control. In the non-stressed conditions potassium ion concentration was significantly higher in the LST genotype. Between the HST and LST plants significant differences were observed on the 6th and 9th day of stress treatment, when a lower value of K^+^ was presented by the salt-tolerant genotype.

### Changes in the transcript abundance of PIP and TIP aquaporins

The transcript level of PIP1;2, PIP2;1, TIP1;1, and TIP2;1 aquaporins under drought, salinity, and cold acclimation was determined using real time qRT-PCR. Material was collected at individual time points, depending on the stress type. For drought, time points were as follows: 0D (before stress treatment), 3-11D (3, 6, 9, and 11 days of water deficit), and RE (10-day irrigation following stress treatment). For salinity time points were as follows: 0NaCl (before stress treatment), and 3-11NaCl (3, 6, 9, and 11 days of stress treatment). For cold stress time points were as follows: 0CA (before stress treatment), and 3-21CA (3, 5, 7, 14, and 21 days of stress application).

Plant response at the transcript level was different depending on a stress treatment. Changes of aquaporin expression was observed in both species. Moreover, the differences in a transcript abundance between highly-tolerant and lowly-tolerant genotypes were additionally observed. Interestingly, the transcript level of TIP2;1 isoform was so low in leaves in both *Festuca* species that its quantitative analysis using qRT-PCR was impossible.

**Fig. 5:**
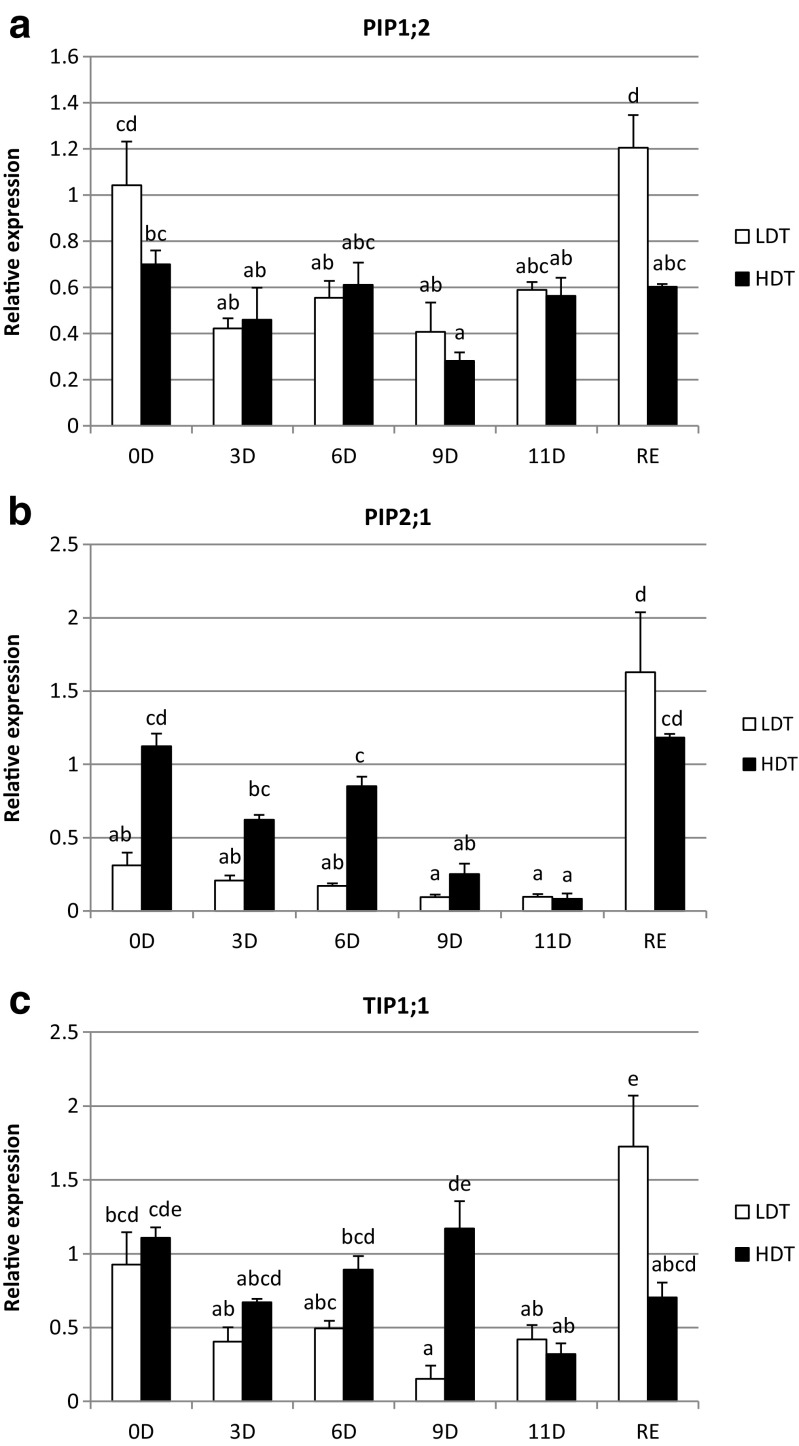
Relative expression profiles of PIP1;2 (**a**), PIP2;1 (**b**), and TIP1;1 (**c**) aquaporins in *F. arundinacea* LDT and HDT genotypes exposed to drought treatment. The transcript levels of actin and ubiquitin were used as reference. Error bars represent the standard errors (SE) of three biological and two technical replicates (*P* < 0.05). Homogeneity groups are denoted by the same letters

Under drought the PIP1;2 aquaporin transcript abundance declined on the 3rd, 6th, and 9th days for the LDT genotype, and on the 9th day in the HDT plant (Fig. 5a). In the control conditions the expression of the gene was similar in both plants. After re-watering both plants exhibited the initial level of PIP1;2 transcript. Water deficit caused significant decrease of PIP2;1 aquaporin abundance on the 9th and 11th days with respect to the control in the HDT genotype (Fig. 5b). In the LDT plant, stress did not influence PIP2;1 transcript abundance. After subsequent re-watering the transcript reached the control level in the HDT genotype, whereas in the LDT it was slightly higher. The level of PIP2;1 transcript differed between genotypes in the initial conditions and on day 6 of drought when it was higher for the HDT plant. The transcript level of TIP1;1 decreased on day 9 in the LDT, and on day 11 in the HDT genotype, compared with the control (Fig. 5c). In the LDT plant after re-watering its abundance was higher than in the non-stress conditions, similar to PIP2;1 isoform. In the control conditions both the LDT and HDT plants showed a similar level of the TIP1;1 expression, whereas under stress conditions the HDT and LDT genotypes differed on day 9 and after rehydration.

**Fig. 6:**
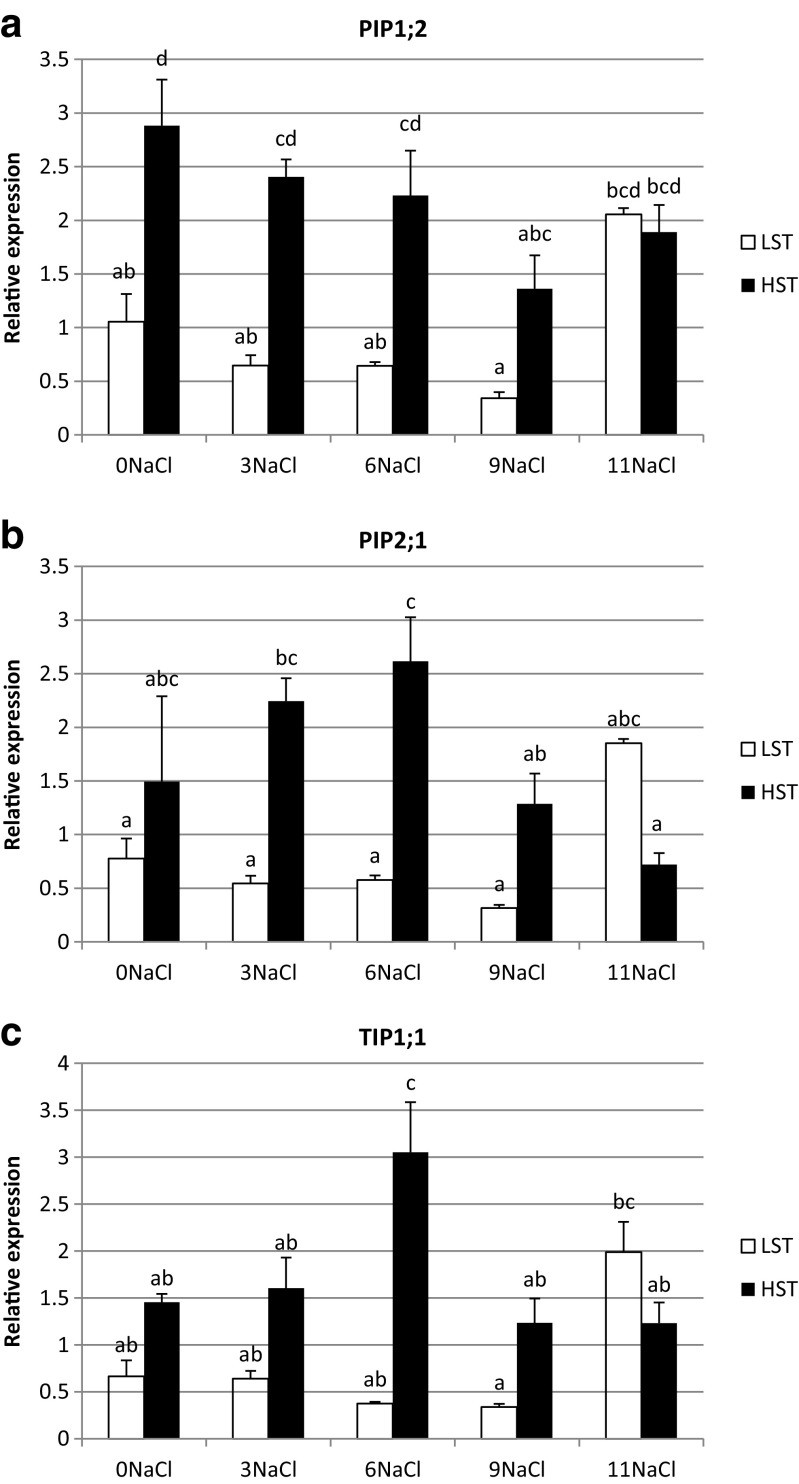
Relative expression profiles of PIP1;2 (**a**), PIP2;1 (**b**), and TIP1;1 (**c**) aquaporins in *F. arundinacea* LST and HST genotypes exposed to salt treatment. The transcript levels of actin and ubiquitin were used as reference. Error bars represent the standard errors (SE) of three biological and two technical replicates (*P* < 0.05). Homogeneity groups are denoted by the same letters

Herein, the impact of salt treatment on aquaporin gene expression is presented (Fig. 6a–c). The salinity did not influence PIP1;2 isoform transcript level in the LST genotype, but caused a decrease of its level on day 9 in the HST plant (Fig. 6a). Differences between genotypes were observed before stress and on the 3rd and 6th days of salinity conditions. The PIP2;1 transcript abundance did not change under salt treatment in both genotypes (Fig. 6b). On the 3rd and 6th day of stress, its higher level was observed in the HST plant, comparing with the LST plant. The increase of TIP1;1 transcript abundance with respect to the control conditions was noticed on day 11 and 6 of NaCl treatment in the LST and HST genotypes, respectively. The initial level of the expression was similar in both genotypes, whereas the differences between them were revealed on the 6th day of stress.

**Fig. 7:**
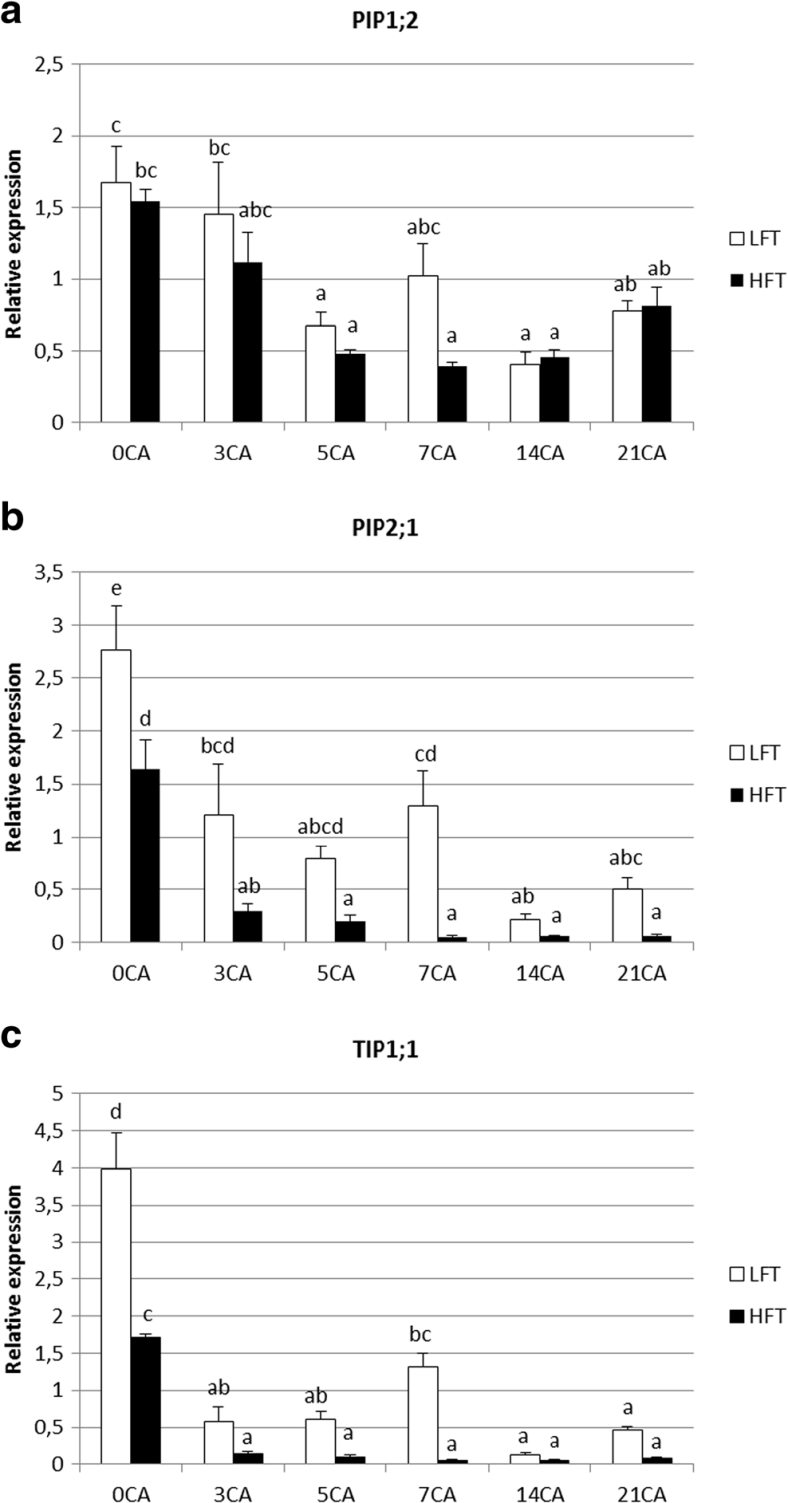
Relative expression profiles of PIP1;2 (**a**), PIP2;1 (**b**), and TIP1;1 (**c**) aquaporins in *F. pratensis* LFT and HFT genotypes exposed to cold treatment. The transcript levels of actin and ubiquitin were used as reference. Error bars represent the standard errors (SE) of three biological and two technical replicates (*P* < 0.05). Homogeneity groups are denoted by the same letters

Cold hardening had the strongest effect on aquaporin gene expression. It resulted in the decline of PIP2;1 and TIP1;1 transcript level during the whole period of cold-hardening both in the HFT and LFT genotypes (Fig. 7b,c). The abundance of TIP1;1 and PIP2;1 aquaporins differed significantly between the genotypes in the control conditions and on day 7 of stress treatment, where it was higher in the LFT genotype. Hardening to cold resulted in some fluctuations in the transcript level of PIP1;2 aquaporin. It dropped on the 5th, 14th, and 21st days of hardening in the LFT genotype, and on the 5th, 7th, and 14th days in the HFT plant compared to the control (Fig. 7a). Gene expression achieved a similar level in both genotypes in non-stressed conditions, and similarly during stress treatment no differences were observed between genotypes at the individual time-points. The exception was day 7 of cold-hardening, with the higher accumulation in the LDT plant, which is similar for the *tip1;1* and *pip2;1* genes.

## Discussion

Aquaporin gene expression profiling is a valuable scientific approach to study the involvement of these proteins in plant response to water relations disturbances accompanying abiotic stresses. Here, the first report on aquaporin contribution in the expression of drought, salt, and freezing tolerance in *F. arundinacea* and *F. pratensis*, the model species among forage grasses, is presented.

Regulation of water status is a complicated process, in which aquaporins fulfill an essential role. In plants, water absorption is tightly linked with CO_2_ assimilation during photosynthesis. There are reports that some aquaporin isoforms belonging to PIPs subfamily have the capacity to facilitate both water and carbon dioxide movement across membranes, suggesting that they may play some important functions in the adjustment of both of these processes under stress conditions (Katsuhara and Hanba 2008; Sade et al. 2010). It was demonstrated that in CO_2_ transport through biological membranes, aquaporins belonging both to PIP1 and PIP2 sub-families are involved (Flexas et al. 2006; Heckwolf et al. 2011; Mori et al. 2014). One such example is the *Arabidopsis* AtPIP1;2 aquaporin and its orthologues in other plant species (Heckwolf et al. 2011; Kaldenhoff 2012; Kaldenhoff et al. 2014). Among PIP2 aquaporins such a function was confirmed for HvPIP2;1 from *Hordeum vulgare* (Mori et al. 2014) and NtPIP1;2 from *Nicotiana tabaccum* (Uehlein et al. 2012). *Arabidopsis* PIP1;2 aquaporin is one of the highly expressed PIP1 aquaporins in leaves (Jang et al. 2004). Aquaporins are the most abundant proteins of the tonoplast, that are connected with a fast osmotic adjustment of the cytoplasm and their role in maintaining osmolality and cell turgor. In *Arabidopsis*, the *Zea mays* and *Oryza sativa* TIPs sub-family is divided into five subgroups according to sequence similarity: TIP1, TIP2, TIP3, TIP4, and TIP5. Localization of TIP2;1 aquaporin in the tonoplast was confirmed for *Arabidopsis* (Liu et al. 2003). Its expression in *Arabidopsis* plants is very high in the vascular system of the shoot but almost undetectable in the roots (Daniels et al. 1996).

Water flux disturbances and photosynthesis rate reduction are the first symptoms in plants after abiotic stress application. One of the primary responses to drought and salt is their root water uptake capacity (root hydraulic conductivity) and water transport inhibition. Both of these responses to osmotic signals are associated with changes in aquaporin gene expression, mainly down-regulation (Martínez-Ballesta et al. 2003, 2006). Low temperature stress can disturb water relations in plants mainly through inhibition of root hydraulic conductivity or cellular dehydration. Under freezing temperatures plant tissues undergo cellular dehydration caused by extracellular ice formation, and consequently water efflux from the plant body might take place. It is thought that both of these phenomena are controlled by aquaporins (Ahamed et al. 2012; Chen and Arora 2014).

In the present study we showed the impact of abiotic stresses treatment on aquaporin genes expression in *F. arundinacea* and *F. pratensis* plants. The genes taken into consideration are members of PIPs and TIPs due to the many reports that aquaporins belonging to these sub-families are regulated by environmental factors and involved in water relations during stresses (Jang et al. 2004; Alexandersson et al. 2005; Zhu et al. 2005; Lian et al. 2006; Hove et al. 2015). The aquaporin family in *Festuca* species has not previously been described and the number of genes from particular sub-families is not known. In these studies we chose isoforms which functions in abiotic stress response were described in other plant species. For our analysis two genotypes with a contrasting level of drought and salt tolerance were selected from *F. arundinacea* and two genotypes with a contrasting level of low temperature tolerance were selected from *F. pratensis* population. The obtained results indicate that PIPs and TIPs aquaporins respond to drought, salinity, and low temperature at the transcript level in *Festuca* species. It concerns the role of aquaporins in maintaining water and photosynthetic homeostasis under abiotic stresses in the forage grasses.

As shown previously by Kosmala et al. (2012), the *F. arundinacea* HDT and LDT genotypes differed between each other in response to water deficit. Other expression profiles obtained for the analyzed aquaporin isoforms can reflect different strategies of drought tolerance represented by the analyzed genotypes. A faster and more significant decrease of RWC during the stress period was observed in the LDT genotype, which also responded to soil water deficit lowering its water uptake. Similarly, in the LDT genotype an increased level of electrolyte leakage (EL) was observed under stress. The return of EL parameters to the initial level after subsequent re-watering indicates that this genotype has a high capacity for membrane injury repair. No EL changes in the HDT genotype under drought suggest it has a more stable membrane system. Drought effects on photosynthesis were also different in both genotypes. Mainly PSII integrity was disturbed in the LDT genotype during drought. After the recovery phase no significant differences between the genotypes were observed. Net photosynthesis, transpiration, or stomatal conductance values achieved the reduced level under water deficit in comparison with the recovery period in both genotypes. During the drought period net photosynthesis and transpiration was higher in the HDT genotype, whereas after rehydration transpiration and stomatal conductance parameters were higher in the LDT.

A lowering of photosynthesis parameters under drought can be associated with a decreased abundance of PIP2;1 transcript observed in the HDT genotype, but not in the LDT, whereas a higher level of PIP2;1 aquaporin in the control conditions in the HDT genotype corresponds with its higher water uptake. Reduction of the PIP1;2 and PIP2;1 transcript level during the long drought duration (9 and 11 days) suggests that in this way the plant reduces membrane water permeability, and limits water loss during stress. A higher level of PIP2;1 isoform observed in the LDT plant during recovery period, in comparison to the control, can lead to the fast achievement of water homeostasis after stress. The contribution of PIP-type aquaporin in the recovery phase was shown in *Jatropha* L. plants subjected to drought stress (Jang et al. 2013). The research performed on MzPIP2;1 aquaporin from *Malus zumi* Mats. indicates that this is one of the most important isoforms involved in water movement from soil to root epidermal cells, and from vessels to parenchyma cells (Wang et al. 2015a, b). Its participation in controlling the efficiency of water absorption and usage under water deficit was demonstrated. The *Arabidopsis* plant with lowered expression of PIP1;2 gene (*Arabidopsis* T-DNA insertion lines) displayed decreased mesophyll conductance (*g*_m_) and photosynthetic activity (reduced net photosynthesis) in comparison to wild plants (Heckwolf et al. 2011).

Similar to PIPs aquaporins, during prolonged drought changes of TIP1;1 transcript level were observed in both analyzed genotypes. TIPs subfamily of aquaporins are localized in the tonoplast of different types of vacuoles. In tobacco suspension cells, overexpression of cauliflower TIP1;1 isoform corresponded with an increase in the cell size (Reisen et al. 2003), whereas overexpression of aquaporin PgTIP1 from *Panax ginseng* increased growth rate in *Arabidopsis* (Peng et al. 2007). This indicates that TIPs are involved in water-driven cell enlargement by modulating the permeability of the tonoplast. The overexpression of PgTIP1 was also shown to change the capacity for not only salt and drought tolerance but also cold acclimation (Peng et al. 2007). There are different reports concerning the influence of the of TIP1;1 isoform loss on plant development. Ma et al. (2004) presented results that suggested that down-regulation of AtTIP1;1 leads to cell and plant death. These authors concluded that this isoform is involved in a vesicle-based metabolite routing between pre-vacuolar compartments and the central vacuole. Beebo et al. (2009) achieved contrasting results showing that knockout mutants did not have phenotypic alterations growing under optimal conditions and AtTIP1;1 loss of function did not cause plant death. A reduced level of TIP1;1 aquaporin transcript noticed in the HDT genotype under drought conditions suggests that this isoform is involved in minimalizing water loss when it is less available. A higher transcript level during recovery period displayed in the LDT genotype could help refill water shortage, as suggested for PIP1;2 aquaporin.

Up- or down-regulation of aquaporin gene expression during salt stress may play roles in limiting initial water loss during the early stage of salt stress, and in assisting the subsequent uptake of water to maintain water homeostasis in high cellular salt conditions. In our experiment the expression of PIP1;2 and TIP1;1 aquaporins during that stress was strongly associated with the salt-tolerance level of genotypes taken to analysis. A salinity treatment caused changes in the transcript level only in the HST plant, a decrease of PIP1;2 level on day 9, and an increase of TIP1;1 abundance on day 6. Moreover, the initial level of PIP1;2 transcript was higher in the HST then in the LST genotype. mRNA-seq analysis performed on *H. vulgare* showed alterations in aquaporin expression under salt treatment (Hove et al. 2015). Among them HvTIP1;1 was up-regulated during stress, in agreement with our studies. Our data is contrary to studies concerning the *Arabidopsis* aquaporin genes. Salt treatment did not influence the expression of PIP1;2 aquaporin in the aerial parts of the *Arabidopsis* plants, but increased its expression significantly in the roots. Transgenic *Arabidopsis* plants with ectopic expression of MzPIP2;1 were more tolerant to slight salt stress (100 mM NaCl), although a higher salt concentration (130 mM) resulted in a negative effect on plant growth, and consequently stress tolerance (weaker growth, shorter root lengths, lighter fresh weight). When grown under 100 mM NaCl stress conditions, transgenic plants had higher K+ content and lower Na+ content then wild-type plants. Under a higher NaCl dose there was the opposite situation, K+ content was lower and Na+ content was higher. The results obtained in our studies revealed an increase and a drop of sodium and potassium content, respectively, in plant biomass in both genotypes during salt treatment. Turan et al. (2010) and Babu et al. (2012) showed that a higher NaCl concentration in the soil resulted in more efficient sodium ions uptake, and a simultaneous decrease of potassium ions uptake into plant biomass as a result of competition between them. Sodium and potassium contents in the fescue plant biomass were highly independent on both genotype and salt treatment duration, which can severely affect ion homeostasis through a handicap of magnesium and calcium uptake, and finally cause deficiency of these elements in the plant (Płażek et al. 2014). It is largely known that salt tolerant and sensitive genotype differ in the accumulation of Na and K ions. Interestingly, the same stress treatment can impact differently on transcript levels of particular genes in different parts of the plant. Jang et al. (2004) showed that the PIP1;1 transcript under NaCl exposure increased both in the aerial parts of the plant and in the roots, whereas the expression of PIP1;2 did not reveal changes in the aerial parts but increased significantly in the roots when grown under salt stress conditions. Overexpression of PIP1;2 aquaporin from *Arabidopsis* in the transgenic tobacco plants did not have the positive effect on plants grown under salt stress, or have the negative effect during drought stress (Aharon et al. 2003). In the analysis of Jang et al. (2004) the abundance of pip1;2 gene transcript was lowered during drought treatment in the aerial parts of plants, but was increased in the roots by salt. It is suggested that these changes may reduce water loss and lead to cellular water conservation during periods with water deficiency. In *Festuca rubra* plants exposed to salt stress (treated with 125 mM NaCl) an increase in the PIP2;1 transcript amount in roots and a decrease in leaves was observed. However, 500 mM NaCl treatment caused a reduction of PIP2;1 expression in roots (Dièdhiou et al. 2009). Both positive and negative influence of enhanced expression of different aquaporin isoforms from PIP and TIP sub-families on salt stress tolerance in transgenic plants were observed (Sade et al. 2014; Wang et al. 2015a, b; Chang et al. 2016; Martins et al. 2017). Both positive and negative influence of enhanced expression of different aquaporin isoforms from PIP and TIP sub-families on salt stress tolerance in transgenic plants were observed (Sade et al. 2014; Wang et al. 2015a, b; Chang et al. 2016; Martins et al. 2017).

Down-regulation of particular aquaporin isoforms observed during low-temperature treatment may be one of the cold acclimation components preventing frost-induced cellular dehydration. An expression profiling at the transcriptional level of 13 *Arabidopsis* PIPs isoforms performed during cold acclimation, and subsequent deacclimation, revealed that most of them, including Pip1;2 and Pip2;1 were down-regulated (Jang et al. 2004). Down-regulation of RcPIP2;1 and RcPIPP2;2 aquaporins transcripts in *Rhododendron catawbiense* Michx. was observed during the seasonal development of cold acclimation in leaves (Peng et al. 2008). Our results show a similar tendency in aquaporin genes expression changes at low temperature. Transcript level of TIP1;1 and PIP2;1 isoforms decreased drastically, especially in the HFT genotype during cold acclimation, suggesting that a lower level of transcriptions is more preferable in the low-temperature conditions.

Wang et al. (2015a, b) reported that the expression of PIP2;1 aquaporin from *Glycine soja* was induced in leaves under cold, drought and salt treatment. Its overexpression in transgenic Arabidopsis plants caused negative effect on salt and drought tolerance and did not influence the plant growth in cold conditions.

Summing up, we made some conclusions concerning the role of aquaporins in abiotic stress tolerance in *Festuca* genus based on the analysis of the accumulation of four PIP and TIP isoforms in leaves and physiological response to cold, drought, and salinity. However, it is important to underline that in plants there is a big number of aquaporin members having different functions both in normal and stress conditions. The expression of the majority of them is organ and tissue-specific and is regulated in different manners depending on stressor type. Results obtained here give us only fragmentary knowledge about involvement of the analyzed aquaporins in acquiring the stress tolerance. Thus, the characterization of the whole aquaporin family in * Festuca* species and expression profiling of all of them (both in upper parts of the plant and the roots) would be required.

## Conclusions

Individual abiotic stimuli affected the transcript abundance of PIP and TIP aquaporin isoforms in *F. arundinacea* and *F. pratensis* species differently.

The reduced level of PIP1;2 aquaporin transcript under advanced drought in *F. arundinacea* was associated with a lowered water uptake in the LDT that resulted in a faster water loss than in the HDT.

The reduced level of PIP1;2 aquaporin transcript during drought was correlated with lowered photosynthesis and gas exchange in the LDT but not in the HDT genotype. It suggests the involvement of this aquaporin in light photosynthetic processes.

A higher level of PIP1;2 and TIP1;1 aquaporin transcripts in the recovery phase with respect to the control conditions suggests that these isoforms can play a role in the water supplementation after stress cessation.

In *F. pratensis* the positive correlation between hardening to cold and decreasing abundance of PIP and TIP aquaporin transcripts was observed.

## Electronic supplementary material

ESM 1 (GIF 277 kb)

High resolution image (TIFF 211 kb)

ESM 2 (DOC 982 kb)

ESM 3 (DOC 44 kb)

ESM 4 (XLS 90 kb)

## Abbreviations

ABS/CS: light energy absorption
ABS/RC: absorbed energy
DIo/CS: dissipated energy flux per CS
DIo/RC: dissipated energy flux
E: transpiration rate
EL: electrolyte leakage
ETo/CS: electron transport flux per CS
ETo/RC: electron transport flux
Fv/Fm: maximum quantum efficiency of PSII photochemistry
Gs: stomatal conductance
HDT: high drought tolerant
HFT: high frost tolerant
HST: high salt tolerant
LDT: low drought tolerant
LFT: low frost tolerant
LST: low salt tolerant
MIP: major intrinsic proteins
NIP: nodulin26 (Nod26)-like intrinsic proteins
PIABS: performance index based on absorbed energy amount
PICS: performance index on a CS basis
PIP: plasma membrane intrinsic proteins
Pn: net photosynthesis rate
RWC: relative water content
SIP: small basic intrinsic proteins
TIP: tonoplast intrinsic proteins
TRo/CS: trapped energy flux per CS
TRo/RC: trapped energy flux
XIP: x ntrinsic proteins
ΦEo: quantum yield of electron transport
Ψo: overall quantum yield between light absorption and the electron transport beyond quinone A
