# Supplementary figures and images for: Abiotic stresses influence the transcript abundance of PIP and TIP aquaporins in Festuca species
Source: J Appl Genet. 2017 Aug 4;58(4):421–35. doi: 10.1007/s13353-017-0403-8 (PMC5655603; doi:10.1007/s13353-017-0403-8)

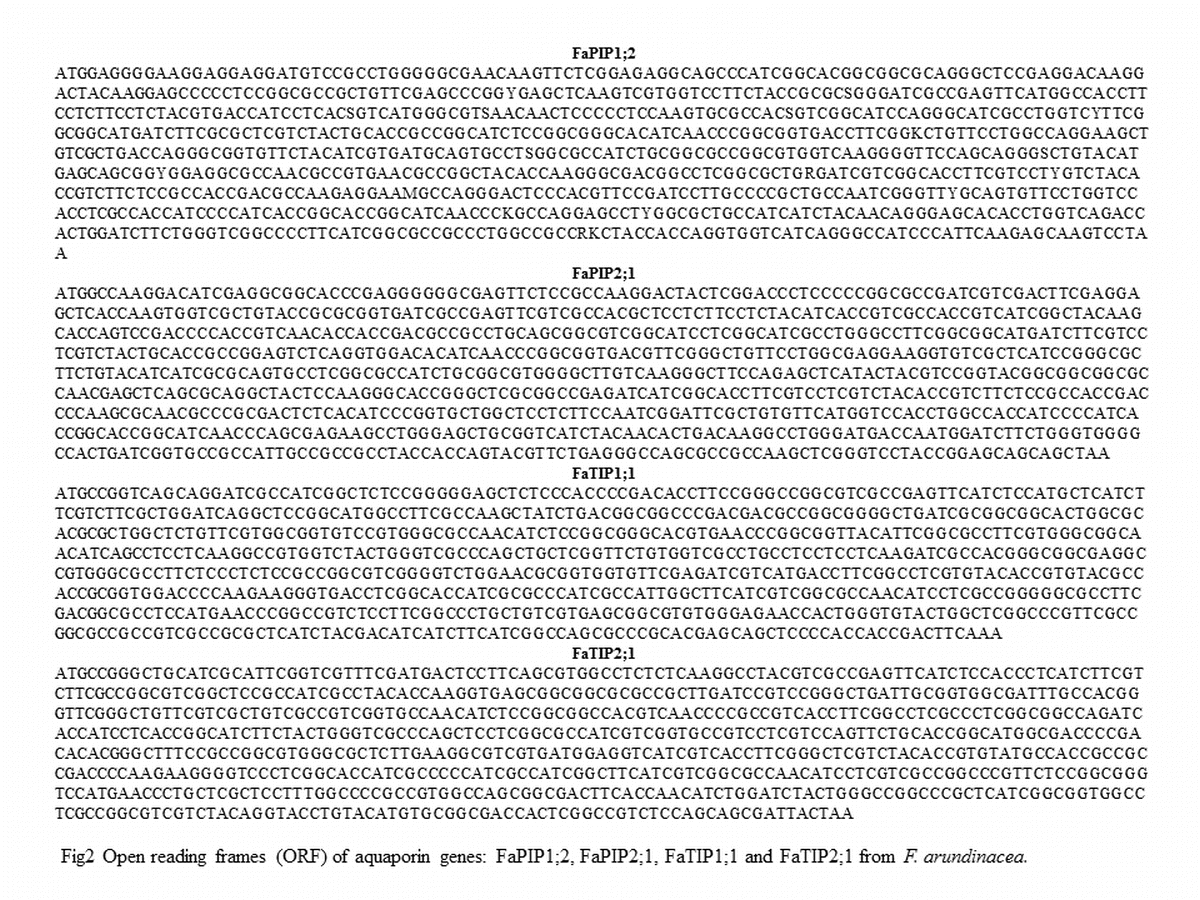

Supplement: Supplementary file 1 — (GIF 277 kb) [file 13353_2017_403_Fig8_ESM.gif]

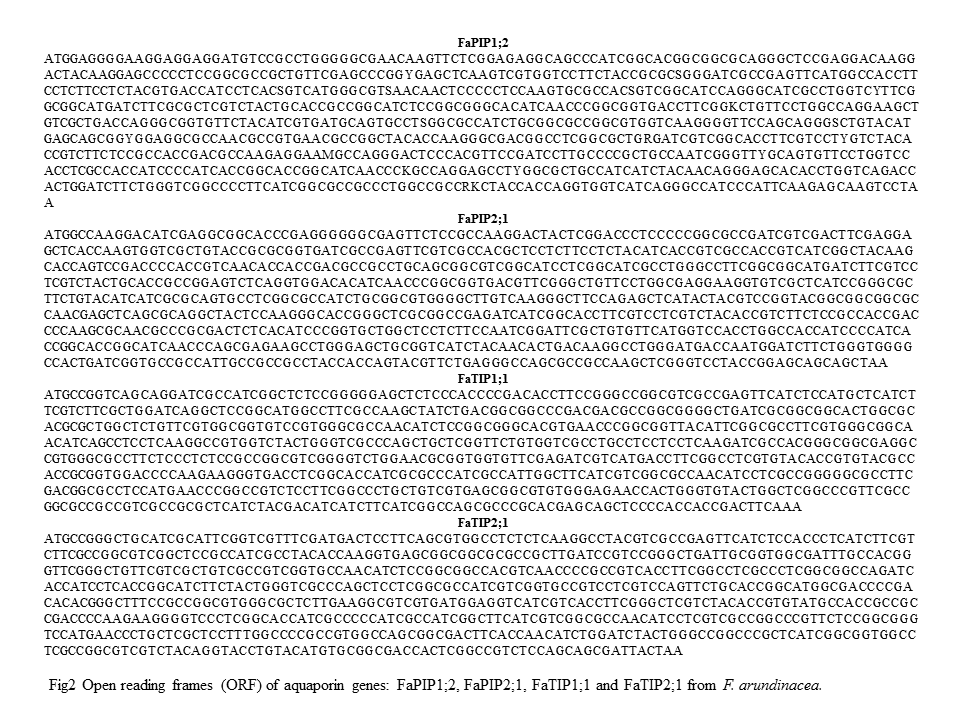

Supplement: Supplementary file 2 — High resolution image (TIFF 211 kb) [file 13353_2017_403_MOESM1_ESM.tif]
