# Supplementary material for: Abiotic stresses influence the transcript abundance of PIP and TIP aquaporins in Festuca species
Source: J Appl Genet. 2017 Aug 4;58(4):421–35. doi: 10.1007/s13353-017-0403-8 (PMC5655603; doi:10.1007/s13353-017-0403-8)
Supplement: Supplementary file 3 — (DOC 982 kb) [file 13353_2017_403_MOESM2_ESM.doc]

“Abiotic stresses influence the transcript abundance of PIP and TIP aquaporins in *Festuca* species”

JAG

Izabela Pawłowicz, Marcin Rapacz, Dawid Perlikowski, Krzysztof Gondek, Arkadiusz Kosmala

Corresponding author: Izabela Pawłowicz, Institute of Plant Genetics, Polish Academy of Sciences, Strzeszynska 34, 60-479 Poznan, Poland, e-mail address: ipaw@igr.poznan.pl


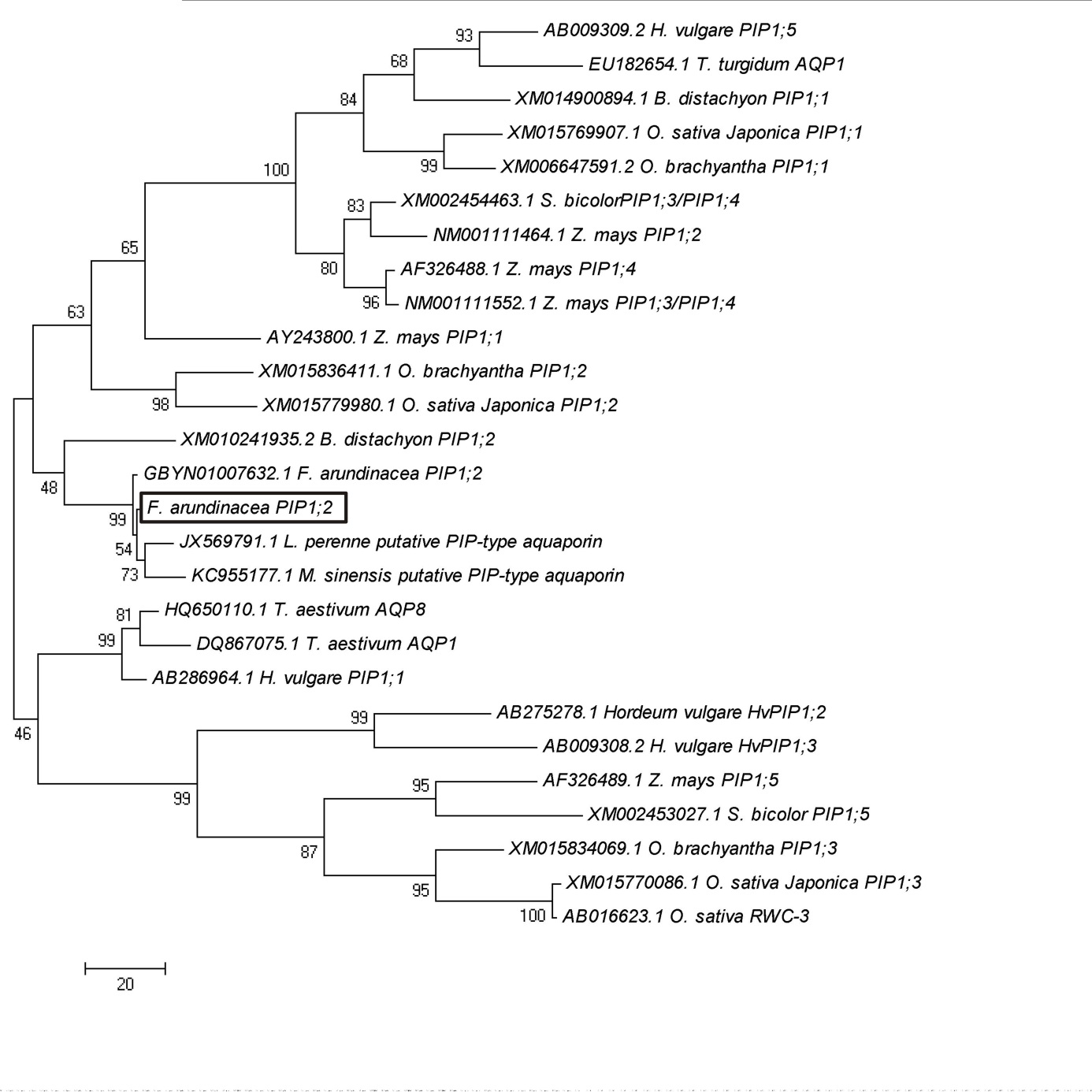


**a.**


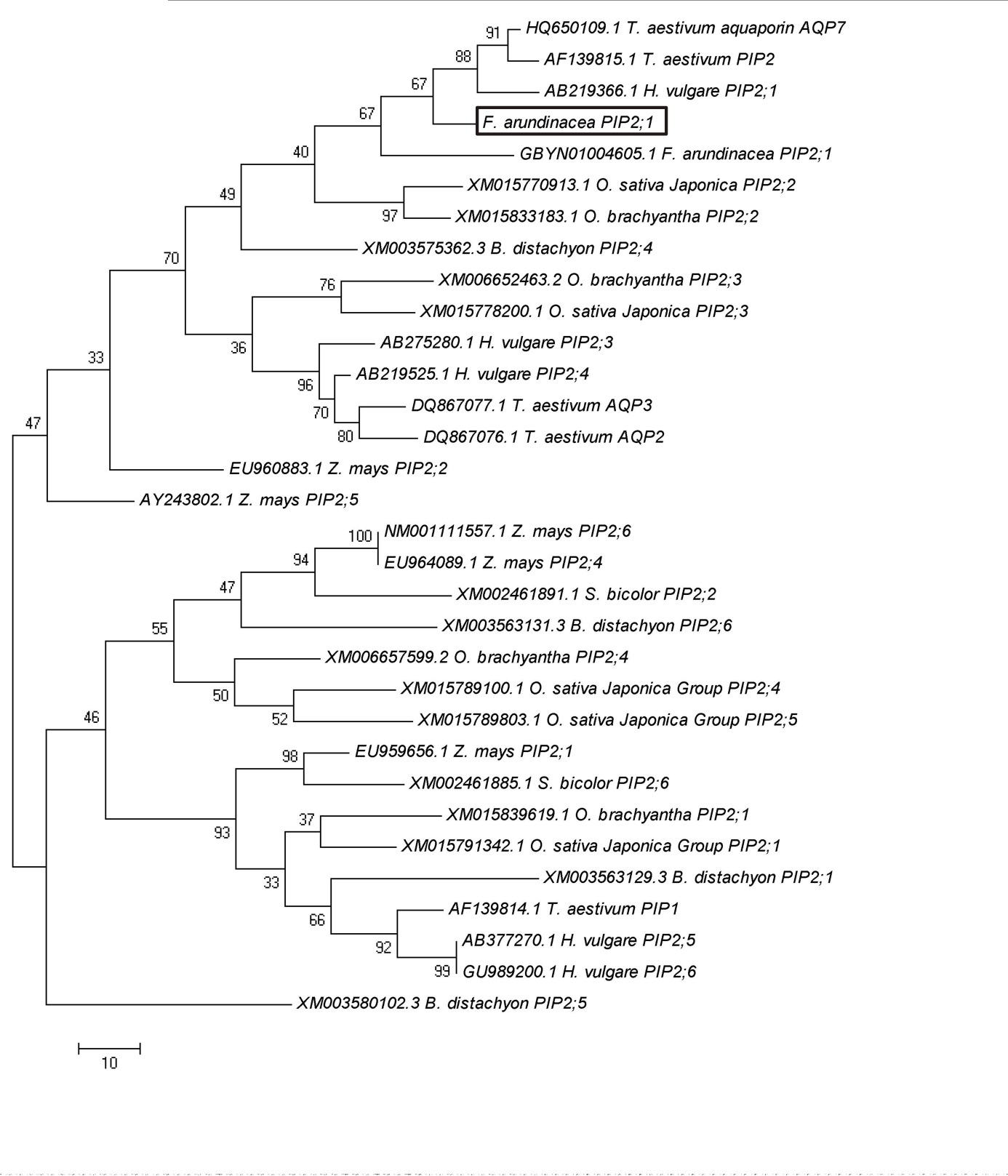


**b.**


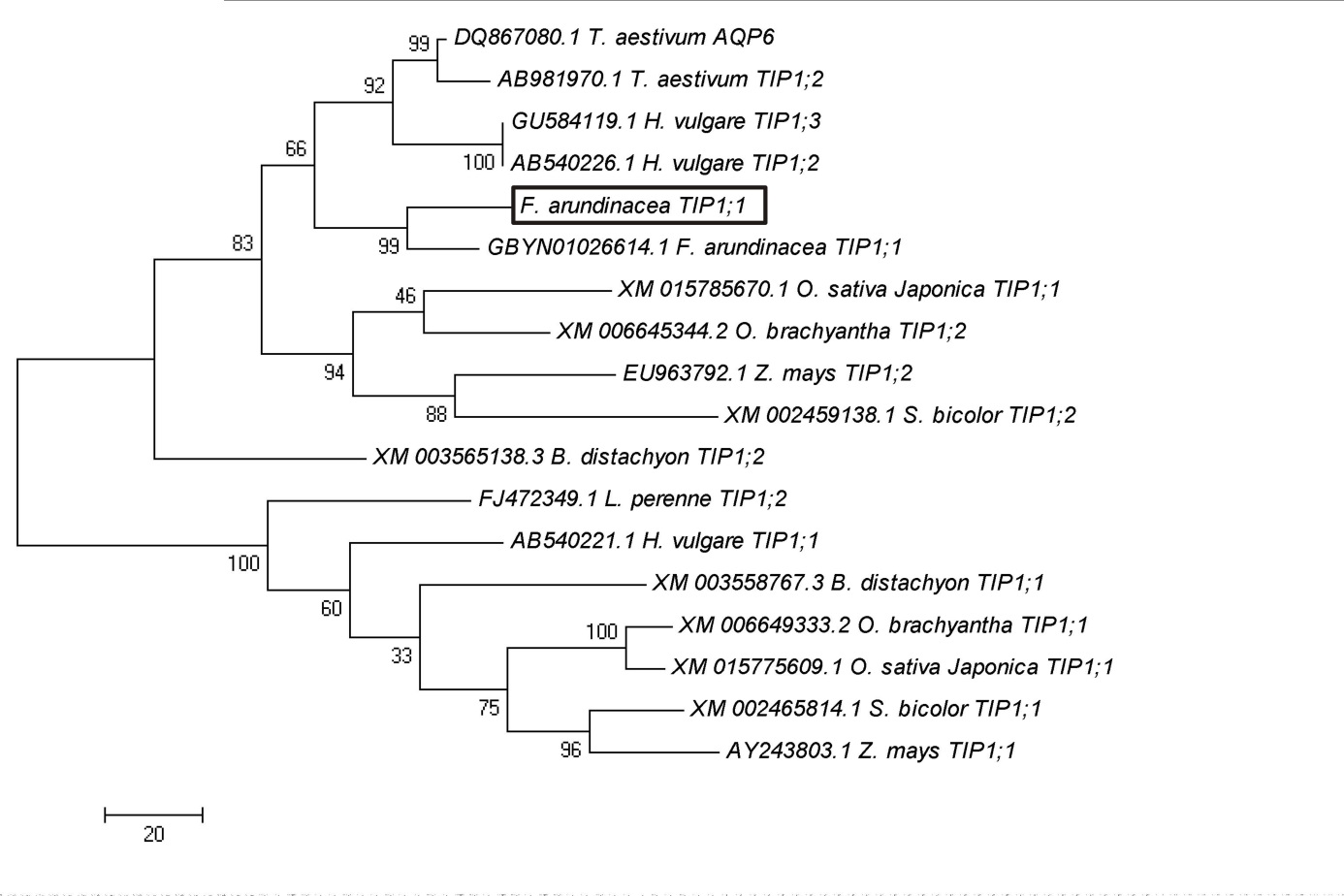


**c.**


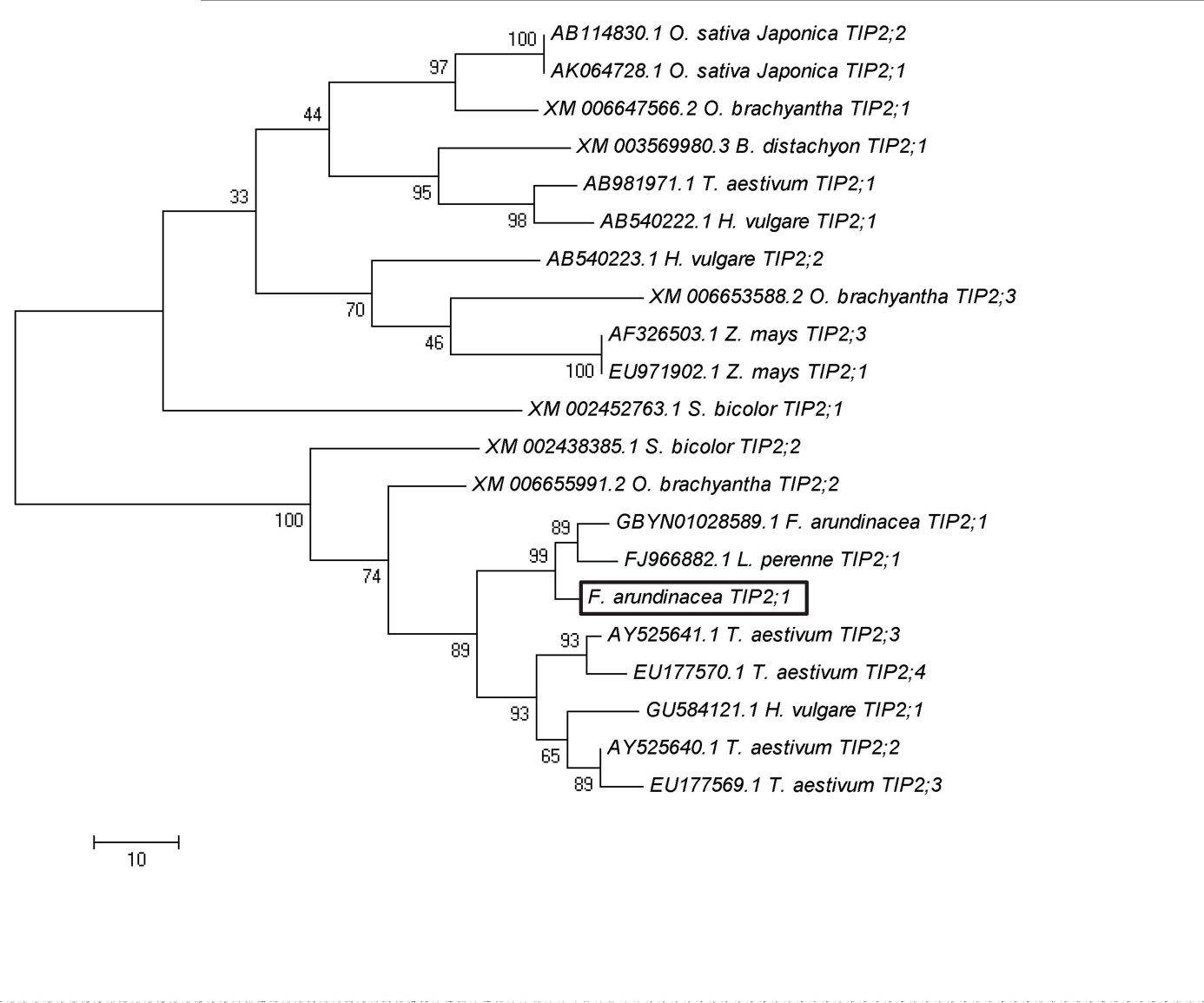


**d.**

**Fig.1** The phylogenetic trees of FaPIP1;2 (a), FaPIP2;1 (b), FaTIP1;1 (c), and FaTIP2;1 (d) aquaporins. The phylogenetic tree was constructed based on Maximum Parsimony approach and bootstrap test (1000 replicates), using MEGA5.2 software. CDS sequences derived from F. arundinacea cv. Kord (FaPIP1;2, FaPIP2;1, FaTIP1;1, and FaTIP2;1) were marked with the frames.

**Fig.1**
