# Supplementary material for: Abiotic stresses influence the transcript abundance of PIP and TIP aquaporins in Festuca species
Source: J Appl Genet. 2017 Aug 4;58(4):421–35. doi: 10.1007/s13353-017-0403-8 (PMC5655603; doi:10.1007/s13353-017-0403-8)
Supplement: Supplementary file 4 — (DOC 44 kb) [file 13353_2017_403_MOESM3_ESM.doc]

“Abiotic stresses influence the transcript abundance of PIP and TIP aquaporins in *Festuca* species”

JAG

Izabela Pawłowicz, Marcin Rapacz, Dawid Perlikowski, Krzysztof Gondek, Arkadiusz Kosmala

Corresponding author: Izabela Pawłowicz, Institute of Plant Genetics, Polish Academy of Sciences, Strzeszynska 34, 60-479 Poznan, Poland, e-mail address: ipaw@igr.poznan.pl

Table 1 Expression stability of reference genes (actin – AKT and ubiquitin – UBQ) under different experimental conditions calculated by BestKeeper. SD: standard deviation; r: coefficient of correlation; p-value: significance level. HST – high salt tolerant genotype, LST – low salt tolerant genotype; HDT – high drought tolerant genotype, LDT – low drought tolerant genotype; HFT – high frost tolerant genotype; LFT – low drought tolerant genotype.

| **Stress** | **Genotype** | **Statistical parameters** | **Reference gene** | |
| --- | --- | --- | --- | --- |
|  |  |  | AKT | UBQ |
| Salinity | HST | *SD* | 0,60 | 0,59 |
|  |  | *r* | 0,815 | 0,899 |
|  |  | *p-value* | 0,001 | 0,001 |
|  | LST | *SD* | 0,68 | 0,76 |
|  |  | *r* | 0,637 | 0,688 |
|  |  | *p-value* | 0,001 | 0,001 |
| Drought | HFT | *SD* | 0,57 | 0,85 |
|  |  | *r* | 0,814 | 0,937 |
|  |  | *p-value* | 0,001 | 0,001 |
|  | LFT | *SD* | 0,40 | 0,44 |
|  |  | *r* | 0,546 | 0,770 |
|  |  | *p-value* | 0,004 | 0,001 |
| Hardening | HFT | *SD* | 0,36 | 0,39 |
|  |  | *r* | 0,86 | 0,89 |
|  |  | *p-value* | 0,001 | 0,001 |
|  | LFT | *SD* | 0,63 | 0,71 |
|  |  | *r* | 0,927 | 0,964 |
|  |  | *p-value* | 0,001 | 0,001 |
